# The OAS-RNase L pathway: insights from experiments of nature

**DOI:** 10.1126/sciimmunol.ads9407

**Published:** 2026-01-09

**Authors:** Danyel Lee, Krishnamurthy Malathi, Tsubasa Okano, Koji Nakajima, Aurélie Cobat, Tomohiro Morio, Jean-Laurent Casanova, Shen-Ying Zhang

**Affiliations:** 1 St. Giles Laboratory of Human Genetics of Infectious Diseases, Rockefeller Branch, The Rockefeller University, New York, NY, USA.; 2 Laboratory of Human Genetics of Infectious Diseases, Necker Branch, INSERM U1163, Paris, France.; 3 Paris City University, Imagine Institute, Paris, France.; 4 Department of Biological Sciences, University of Toledo, 2801 West Bancroft Street, Toledo, OH 43606, USA.; 5 Department of Pediatrics and Developmental Biology, Graduate School of Medical and Dental Sciences, Tokyo Medical and Dental University (TMDU), Tokyo, Japan.; 6 Lymphocyte Nuclear Biology, NIAMS, NIH, Bethesda, MD 20892, USA.; 7 Department of Pediatrics, Necker Hospital for Sick Children, Paris, France.; 8 Howard Hughes Medical Institute, The Rockefeller University, New York, NY, USA.; 9 Howard Hughes Medical Institute, Rockefeller University, New York, NY 10065, USA

## Abstract

The 2’-5’ oligoadenylate synthetases (OASs) are type I IFN-inducible enzymes that, with RNase L, have been studied in the context of their coupled action as antiviral effectors. RNase L degrades host and viral ssRNA, impacting diverse cellular processes including translational arrest, interferon response and apoptosis, all of which are thought to restrict viral replication. Recent studies of recessive inborn errors of human OAS1, OAS2 and RNase L, however, revealed that for SARS-CoV-2 infection, the main protective action of this pathway in natura may be through restricting phagocyte-driven post-viral inflammation rather than restricting early viral replication in the respiratory tract. This finding is consistent with the identification of gain-of-function *OAS1* mutations in humans with autoinflammation also driven by myeloid cells. Here, we retrace investigation of the OAS-RNase L pathway, focusing on these recent in natura studies in humans that reposition the pathway as a determinant of the inflammatory response under natural conditions of infection.

## One-sentence summary

Human monogenic deficits or excesses in the OASs and RNase L, which reposition the OAS-RNase L pathway as a regulator of inflammation, are reviewed.

## Introduction

The discovery of interferon (IFN) in 1957 quickly led to the identification of various IFN-inducing and IFN-responsive pathways, and many IFN-regulated genes (IRGs) (1). In humans, type I IFNs are antiviral and type II IFN is a macrophage activation factor (2). Type III IFNs contribute to antiviral responses and appear to have immunomodulatory properties, but their non-redundant roles during human infections have yet to be deciphered (2). Type I and III IFNs are secreted by cells upon activation of a nucleic acid sensor following the binding of RNA or DNA of viral (viral genome or viral replication intermediates) or cellular (self-DNA or RNA, by-products of viral infection) origin (3, 4). Type III IFN receptors are expressed exclusively by epithelium and endothelium cells, whereas type I IFN receptors are ubiquitously expressed throughout the human body (1). While most studies on IRGs have focused on type I IFN-stimulated genes (ISGs), these have considerable overlap with type III IFN ISGs, as the receptors of both IFNs signal through the same JAK-STAT pathway (1). Depending on cell type, at least hundreds, if not thousands, of ISGs can be induced by type I IFNs (5). One major branch of ongoing effort has focused on the identification and characterization of ISG-encoded proteins that possess antiviral effector functions. The best-known examples include ISG15, PKR, Mx1, and the 2’-5’ oligoadenylate synthetases (OASs), which were discovered in the late 1970s and have since been studied as host effector ISGs (6–8).

In humans, monogenic deficiencies of type I IFN production or response lead to impaired restriction of viral infection in certain organs, particularly the brain and the lungs, resulting in severe acute viral inflammation (2, 9, 10). Conversely, Mendelian interferonopathies lead to excessive IFN responses, resulting in autoinflammation (4). The efforts of human immunology in recent years have, thus, elegantly confirmed the initial finding of the classic IFN immunology field that type I IFN responses are nonredundant for host antiviral defense against certain viruses in natura. However, human patients with inborn errors of type I IFN immunity present only a narrow range of viral infections, suggesting a certain redundancy of global type I IFN responses against many other viral infections.

This apparent redundancy subsequently raised questions about the specific roles of individual antiviral effector ISGs in natural conditions of infection (11). Inborn errors of immunity of the classic antiviral effector ISG *MX1* were found to underlie critical pneumonia following infection with the highly virulent avian influenza virus (12). Meanwhile, inborn errors of immunity of another classic antiviral effector ISG, *ISG15*, were shown to underlie mycobacterial disease due to impaired IFNγ-mediated immunity and atypical interferonopathy due to unrestricted type I IFN immunity in humans (13). Inborn errors of immunity of the OASs and ribonuclease L (RNase L), the only known effector of OASs, have recently been identified in humans with unsuspected but compelling phenotypes, providing new perspectives on the biology of the OAS-RNase L pathway (14, 15). Here, we retrace investigation of the OAS-RNase L pathway, focusing on recent in natura studies in humans that have positioned the pathway on the ridge between infection and inflammation.

## The OAS-2-5A-RNase L system

**Figure 1.**
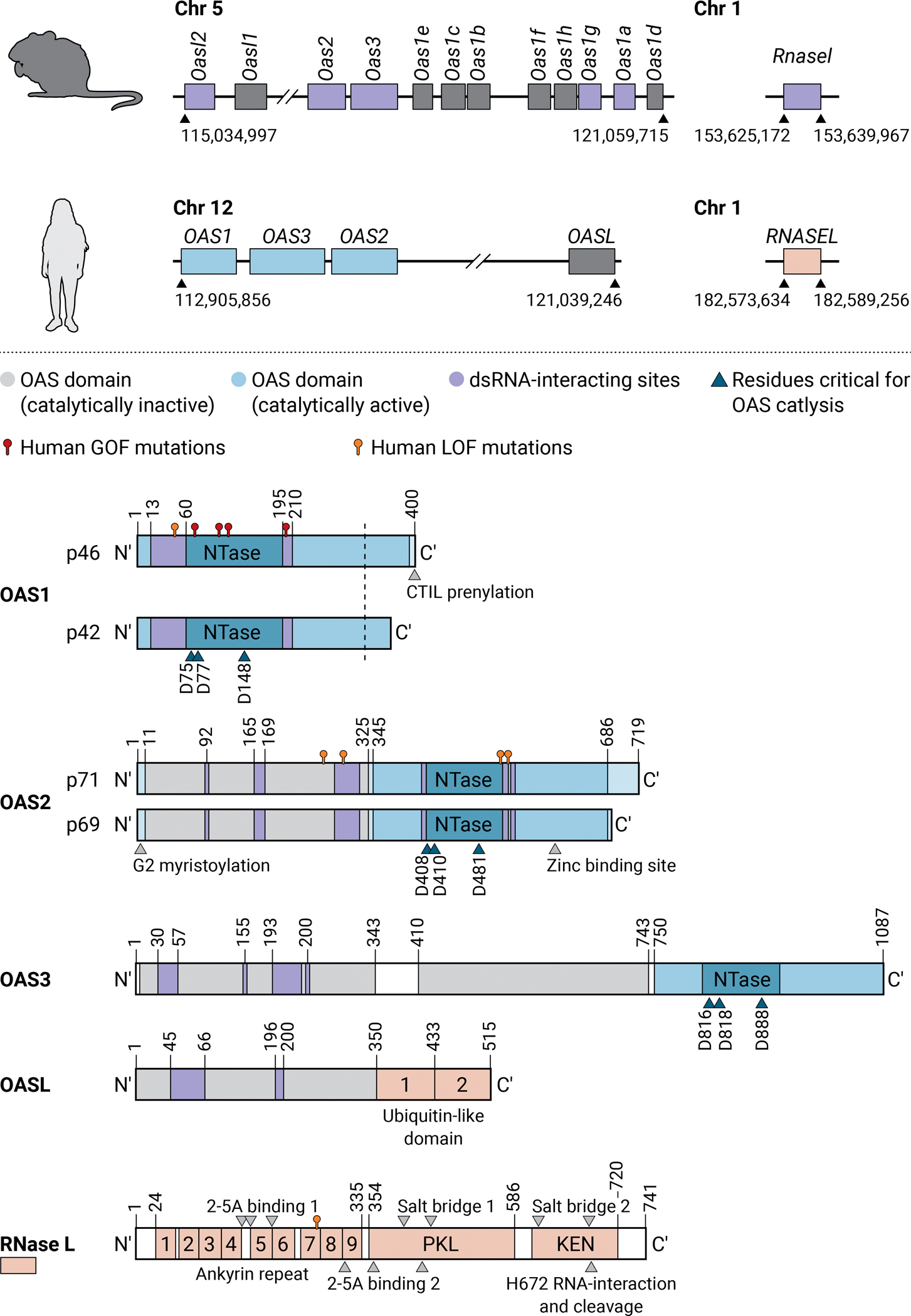
Human and mouse *OAS* and *RNASEL* genes and their protein products in humans. Schematic representation of the *OAS* and *RNASEL* gene loci in mice and humans (above). Loci coding for catalytically inactive OASs are in dark gray. The human protein products are also shown (below). The different sites for interaction with RNA (purple), catalytic activity (dark blue arrows), and dimerization or subcellular localization (gray arrows) are indicated for the OASs and RNase L. Residues in RNase L that interact with 2–5A are also indicated by gray arrows. Pathogenic mutations described in humans are indicated with pins. Red pins indicate gain-of-function (GOF) mutations and orange pins indicate loss-of-function (LOF) mutations. NTase – nucleotidyltransferase domain; PKL – protein kinase-like domain; KEN – kinase extension of the nuclease domain; 2–5A – 2’-5’-linked oligoadenylate.

The OASs are proteins that recognize cytosolic dsRNAs and produce 2’-5’ linked oligoadenylates (2–5A) upon their binding. 2–5A then activates the latent endoribonuclease, RNase L, that then serves as an endoribonuclease that cleaves exogenous (e.g. viral genome or RNA) and endogenous single-stranded RNAs (ssRNAs). OASs belong to an ancient family of template-independent RNA polymerases and are thought to have evolved from a common ancestral gene through gene duplication and domain coupling driven by positive selection (16–20). The human genome contains three genes encoding catalytically active OASs, *OAS1*-*3*. Through alternative splicing, these genes can generate 10 different transcripts encoding the corresponding predicted isoforms: OAS1 (p42, p44, p46, p48, and p52), OAS2 (p69 and p71), OAS3 (p100)(21). The endogenous protein isoforms identified in human cells and shown to be active are OAS1 p42 and p46, OAS2 p69 and p71, and OAS3 p100 (22–24). The basal expression level of the OASs differs between cell types, but all three human OASs can be further induced by type I, II, and III IFN (15, 21, 25, 26). The human genome also contains a gene encoding a catalytically inactive OASL, which generates three isoforms, only the longest of which has been characterized (27, 28) (Fig. 1).

The human OAS proteins contain one (OAS1), two (OAS2), or three (OAS3) nucleotidyltransferase (NTase) domains arranged as tandem repeats, in which only the C-terminal unit is catalytically active to produce 2–5A but all units are necessary for catalysis (29–32)(Fig. 1). Despite shared structural patterns, the OASs differ in their capacity to bind dsRNA of different lengths and modifications, their capacity for multimerization, their subcellular compartmentalization, and the species of 2–5A that they produce (22, 33). Binding to dsRNA is required to induce the conformational change necessary for catalytic activity of the OASs (31, 34). The OASs bind dsRNA at a conserved site with positively charged amino acids on the surface of the protein with low sequence-specificity, although certain motifs seem to better activate OAS1 and OAS3 (33–37). Their diverging optimal dsRNA length for catalytic activity is in part due to the number of OAS domains that serve as a molecular ruler, but also their multimerization (32, 33, 37–39). For example, OAS2 was recently demonstrated to form a stable homodimer in an autoinhibitory state, avoiding activation by shorter dsRNAs (37, 40–42). The different subcellular localization of the OASs to the cytosol or the endomembrane system (22, 43), dictated by different posttranslational modifications (prenylation of OAS1 p46 and myristoylation of OAS2), can also influence their function by strategically positioning them at privileged sites of virus replication, but also, in the case of OAS2 and OAS3, constraining their enzymatic activity (22, 37, 44–47).

Following generation of 2–5A that is trimeric or of higher order by OASs, these messengers activate the latent endonuclease RNase L (29, 48, 49). RNase L is expressed in all cell types as an autoinhibited inactive form (50–54). RNase L is thought to have functionally coupled with the OASs in the jawed vertebrates and coevolved with them (20). More generally, the OAS-RNase L pathway seems to be part of a unified nucleotide-based signaling involved in diverse biological processes across living organisms (55). Indeed, parallels between the dsRNA-sensing OAS-RNase L pathway and the dsDNA-sensing cyclic GMP-AMP synthase (cGAS)-STING pathway exist, although no direct phylogenetic connection has been made (34, 56). The domain structure of RNase L includes an N-terminal ankyrin repeat (ANK), followed by a protein kinase-like domain (PKL) and a C-terminal kinase extension of nuclease (KEN) domain (Fig. 1). 2–5A binds two ANK and one PKL domain, and this, together with the binding of nucleotide to the PKL active site, induces the formation of an intertwined closed dimeric active form of RNase L. The active RNase L then cleaves exogenous (e.g. viral genome or RNA) and endogenous ssRNAs after UpU and UpA dinucleotide sequences (57–60). Non-enzymatic functions of RNase L have also been described (61), but, to date, the best-established major function of RNase L is the cleavage of ssRNAs (57, 62–65).

The downstream antiviral effects of RNase L activation, attributed either to a direct nucleolytic function of RNase L on ssRNA or indirect mechanisms involving the activation of other dsRNA-sensing pathways by RNase L cleavage products, have been intensively studied at the molecular and cellular level (66, 67). These include a cascade of cellular signaling to induce cell-intrinsic and paracrine responses implicated in virus restriction, host cell cycle, and proinflammatory cytokine production. Recently, it has been shown that, like cGAMP, 2–5A can also be imported, exported, and transferred directly to neighboring cells through gap junctions, allowing a preemptive initiation of these processes(68). In this review, we focus primarily on the four molecules of the human OAS-RNase L pathway (OAS1, OAS2, OAS3, RNase L) and their concerted action, in the light of recent genetic findings in humans.

## The OAS-RNase L pathway in virus restriction, cell death and immune regulation

The antiviral effects of the OASs and RNase L has been examined against many viruses in overexpression assays in vitro, mostly in mouse fibroblasts or human epithelial cell lines (Table S1) (37, 44–47, 67, 69–80). In overexpression conditions, OAS1–3 or RNase L could restrict a variety of RNA viruses, including SARS-CoV-2 (37, 44, 45, 47, 67, 71, 72, 78–80). Studies in OAS- or RNase L-deficient cells, however, revealed a narrower contribution of the pathway in virus restriction (Table S1). Except for encephalomyocarditis virus (EMCV) and Sendai virus, for most viruses tested, the RNase L pathway has appeared redundant for protection conferred by type I or type II IFN (15, 67, 81–83). In vivo mouse forward genetics have robustly demonstrated that Oas1b is a major determinant of flavivirus resistance (46, 84, 85), but its antiviral activity is 2–5A-, and therefore, RNase L-independent. The majority of in vivo reverse genetic studies have been performed using RNase L-deficient mice challenged with various viruses from diverse genera (67, 86). RNase L-deficient mice generally display a milder phenotype than mice lacking type I IFN receptors, and IFN only partially rescues the phenotype of RNase L-deficient mice.

Later studies highlighted the organ-, and ultimately, cell type-specific activity of the OAS-RNase L pathway against certain viruses (i.e., herpes simplex virus 1 (HSV1) and mouse hepatitis virus (MHV)). RNase L is essential for the restriction of MHV replication in the liver but not in the brain, and was found to specifically act in macrophages and microglia, in which basal *Oas* and *Rnasel* expression levels were highest (81, 87, 88). Thus, the in vivo functions of the OAS-RNase L pathway in mice have proven to be more complex than simply exerting IFN-induced antiviral effector activity. Activity may differ between cell types, with myeloid cells of particular interest, probably due to their high basal level of *Oas*/*Rnasel* expression and the nature of the pathogen triggering the response. Yet, strategies for evading the OAS-2–5A-RNase L system have been described for a number of viruses (e.g. influenza A virus (IAV), group A rotavirus, coronaviruses), suggesting that this system has potent antiviral capacities that viruses evolved to overcome (reviewed in (89)).

Given its inhibitory effect on protein synthesis, the role of the OAS-2–5A-RNase L system in cell proliferation and cell death was suggested very early, notably as a mediator of the IFN-induced anti-proliferative effects (29). In vitro, activation of the OAS-RNase L pathway is pro-apoptotic, an effect that has been suggested to represent an antiviral strategy, as well as have implications for the survival of cancerous cells (90–95). RNase L-mediated ribosomal RNA degradation and RNase L cleavage products induce autophagy via PKR activation and the ribotoxic stress response, while sustained or excessive RNase L activation induces apoptosis (86, 90, 91, 96, 97). RNase L-deficient mice do not spontaneously display notable abnormalities except for thymic hyperplasia (86). A recent study showed that MC38 tumors secreting high levels of 2–5A grew faster when implanted in RNase L-deficient mice, an effect dependent on host RNase L as well as tumor OAS proteins and the 2–5A transporter ABCC10, revealing a role for RNase L-mediated antitumor immunity in response to tumor-secreted 2–5A (68). Conversely, in Adar1-deficient mice, concomitant RNase L deletion did not rescue lethality or weight loss, despite prior reports that RNase L is the major driver of cell death in an in vitro human cancer cell line model of ADAR1 deficiency (92, 94, 98). Collectively, these observations suggest that the contribution of OAS-RNase L-mediated cell death in human physiology and disease is likely context-dependent.

During an immune response, the OAS-RNase L pathway can exert both proinflammatory and anti-inflammatory activity. Despite the broad substrate range for RNase L, certain transcripts seem to escape RNase L-mediated decay, in particular some ISGs (e.g. *OAS2*, *IFIH1*, *DDX58*, *DHX58*) and antiviral and proinflammatory cytokines (e.g. *IFNB1*, *IFNL1/2*, *IL6*, *CXCL2*)(99–103). In addition, RNase L has been shown to sequester *IFNB1* mRNA in the nucleus, and OAS1 to sequester *IFNB1* mRNA in a RNase L-independent manner (46, 104). Conversely, RNase L cleavage RNA products can activate the RIG-I/MDA5-MAVS pathway and, in human and mouse myeloid cells, the NLRP3 inflammasome via DHX33 (105)*(106)*. However, this secondary stimulation of additional dsRNA-sensing pathways appears to be quenched by other cellular pathways, such as the RNA exosome (107). Notably, the immune regulatory effect of the OAS-RNase L pathway activation differs depending on the cell types. In mouse embryonic fibroblasts, genetic deletion of RNase L appears to ablate IFN-β secretion in response to poly(I:C) or EMCV infection in vitro, while in mouse macrophages and human monocytic cell lines, OAS-RNase L deficiency results in enhanced IFN-β production (15, 108, 109). This is possibly due to different levels of basal expression of OASs and RNase L or possibly of their regulators in the different cell types (15, 108, 109). Thus, the OAS-RNase L pathway can be antiviral, proapoptotic and pro- or anti-inflammatory, depending on the cellular context.

## Human population genetics of the *OAS*s and *RNASEL*

In humans, the four *OAS* genes (*OAS1*, *OAS2*, *OAS3*, *OASL*) lie in a region of about 8 megabase pairs on chromosome 12, whereas *RNASEL* is located on chromosome 1 (Fig. 1). The human *OAS1*, *OAS2*, *OAS3*, and *RNASEL* genes contain 7, 10, 16 and 8 exons, respectively. The promoter of the *OAS1* gene has an interferon-stimulated response element (ISRE), another interferon regulatory factor element (IRF-E), and a gamma interferon activation site element (GAS); that of *OAS2* has two ISRE sites, two other IRF-E sites and sites for NFκB; and the promoter region of *OAS3* contains two ISREs, three GASs, and an NFκB binding site (21, 25, 110). *RNASEL* is ubiquitously expressed under the control of a wide range of tissue-specific and general promoters (52). However, *RNASEL* expression is subject to strict posttranscriptional regulation mediated by binding of AU-rich elements in the 3’-UTR to Human antigen R (HuR) (111). After translation, RNase L is susceptible to ubiquitination, which can lead to its rapid degradation via the ubiquitin-proteasome pathway (112).

All four genes (*OAS1*, *OAS2*, *OAS3* and *RNASEL*) are highly conserved across species and in humans. *In silico* analysis at the population level has predicted a gene damage index (GDI) and a consensus negative selection score (CoNeS) of 4.22 and 2.24, respectively, for OAS1, 1.0 and 0.79 for OAS2, 5.65 and 1.45 for OAS3, and 8.78 and 0.66 for RNASEL, consistent with an autosomal recessive (AR) predisposition to life-threatening infectious diseases (113, 114). In the gnomAD database (v4.1.0), the largest human exome/genome sequencing database currently available (115), the ratios for “observed/expected number of predicted loss-of-function (pLOF) variants” for the four genes are 0.57, 0.88, 0.95 and 0.61, respectively, suggesting that the four genes are not under negative selection. However, only 1, 1, 1 and 2 relatively rare pLOF variants (minor allele frequency <0.01) are found in the homozygous state in 4, 1, 1 and 23 individuals, respectively, of the 807,162 individuals for whom exome/genome data are available. The estimated cumulative frequency of homozygous carriers of pLOF variants at the three loci is, therefore, below 0.0001 in the general population, suggesting that AR deficiencies of the four genes might underlie disease. Hence, given the unique evolution of the *OAS* and *RNASEL* genes across species, the human *OAS* and *RNASEL* genes are probably determinants of certain diseases or health conditions.

## Association studies of the human OAS-RNase L pathway in infectious diseases and cancer

Over the last two decades, multiple candidate gene-based association studies have evaluated the link between common polymorphisms of *OAS* genes and the outcome of a number of viral infections in humans. Several single nucleotide polymorphisms (SNPs) in *OAS1*, *OAS2*, *OAS3* and *OASL* have been reported to be significantly associated with various viral phenotypes (reviewed in (114), such as West Nile virus (WNV) seroconversion and encephalitis, Dengue fever, tick-borne encephalitis virus-induced disease, hepatitis C virus (HCV) progression, or severe acute respiratory syndrome (SARS) (116–119). However, the results of these association studies were not always consistent, with odds ratios (OR) ranging from 1.35 to 9.79 for risk alleles, and from 0.09 to 0.76 for protective alleles, illustrating the limitations of candidate gene-based association studies in general (116–119). Moreover, most studies were performed on relatively small samples, consisting of no more than 330 disease cases, and did not account for population stratification or multiple testing. The results obtained are, therefore, inconclusive. Recent advances in large-scale genotyping technology have made it possible to explore the full human genome for common infectious disease-associated variants in an unbiased manner. Genome-wide association studies (GWAS) have identified many associations between other common human genetic variants and infectious diseases (120). However, the results of GWAS for HCV-related phenotypes (121–124) and WNV infection (125) did not confirm the previously reported association with *OAS* polymorphisms.

More recently, during the COVID-19 pandemic, common polymorphisms of the *OAS1/2/3* gene cluster were consistently reported to be associated with COVID-19 severity, with ORs ranging from 0.78 to 0.94 for protective alleles and from 1.19 to 1.33 for risk alleles, in GWAS on individuals mostly of European ancestry (71, 126–129). The associated polymorphisms belong to a haplotype derived from Neanderthal introgression (130), for which population genetic signatures of positive selection have been detected in the European population (131). These GWAS showed, in particular, that common variants at the *OAS1* locus were associated with COVID-19 severity, with ORs between 0.78 and 0.94 (45, 127–129, 132, 133). One of the candidate causal variants in this region is the *OAS1* splice variant rs10774671. The reference G allele, inherited from the Neanderthals, is present in about 37% of the general human population and has been shown to be associated with mild protection against severe forms of COVID-19 (OR ~0.9) (127, 129, 132, 133). This variant is also associated with protection independently of gene flow from the Neanderthals in people of African ancestry (OR ~ 0.9) (128). The G allele encodes the OAS1 p46 isoform, whereas the alternative A allele encodes the p42 isoform. In vitro studies have shown that C-terminal prenylation of p46 allows OAS1 to localize to organelles in which SARS-CoV-2 replicates, and thus enhances OAS1 inhibition of SARS-CoV-2 replication in an RNase L-dependent manner in overexpression models (Table S1) (44, 45, 72). However, complete loss of OAS1 did not affect the protection against SARS-CoV-2 conferred by type I IFN (15, 45, 71, 78–80). Thus, despite the clear contribution of OAS1 to host defense against SARS-CoV-2 that is detectable at the population level, the biological explanation for the association of the variants detected by GWAS with COVID-19 severity warrants further clarification.

Similarly, the expression levels of the OASs have been associated with the prognosis of various cancers. Gene expression profiles of the OASs have been suggested to be useful biomarkers of interferon signaling in the tumor or its microenvironment that could modulate tumor progression, immunosuppression, treatment response, and metastasis (68, 134–139). A more direct role of the OAS-RNase L pathway in cancer development and progression has been suggested by its pro-apoptotic effects or potential to amplify interferon signaling (68, 140). *RNASEL* specifically has been proposed as a strong candidate gene for hereditary prostate cancer (*HPC1*). Statistically significant linkage was found between hereditary prostate cancer and the chromosome 1q23–25 locus, where *RNASEL* lies, though this has not always been reproducible. The linkage was strongest in families with at least five afflicted family members, early age (<65 years) diagnosis, and male-to-male transmission (peak heterogeneity logarithm of the odds score (HLOD) 2.25, P=0.001). Several case-control studies have found germline pLOF mutations (E265X, E262X, M1I, 471delAAAG) as well as two missense mutations (R462Q, D541E) leading to functionally hypomorphic RNase L proteins with often conflicting ORs varying between 0.54 and 3.07, depending on the study and the genotype (95). The true impact of the different germline *RNASEL* variants in susceptibility to prostate cancer remains unclear. Whether additional somatic mutations in RNASEL or related genes in prostate tissue or its surrounding microenvironment could further contribute to the phenotype expression has not been investigated.

## OAS1 gain of function genotypes in humans: autoinflammation

**Figure 2.**
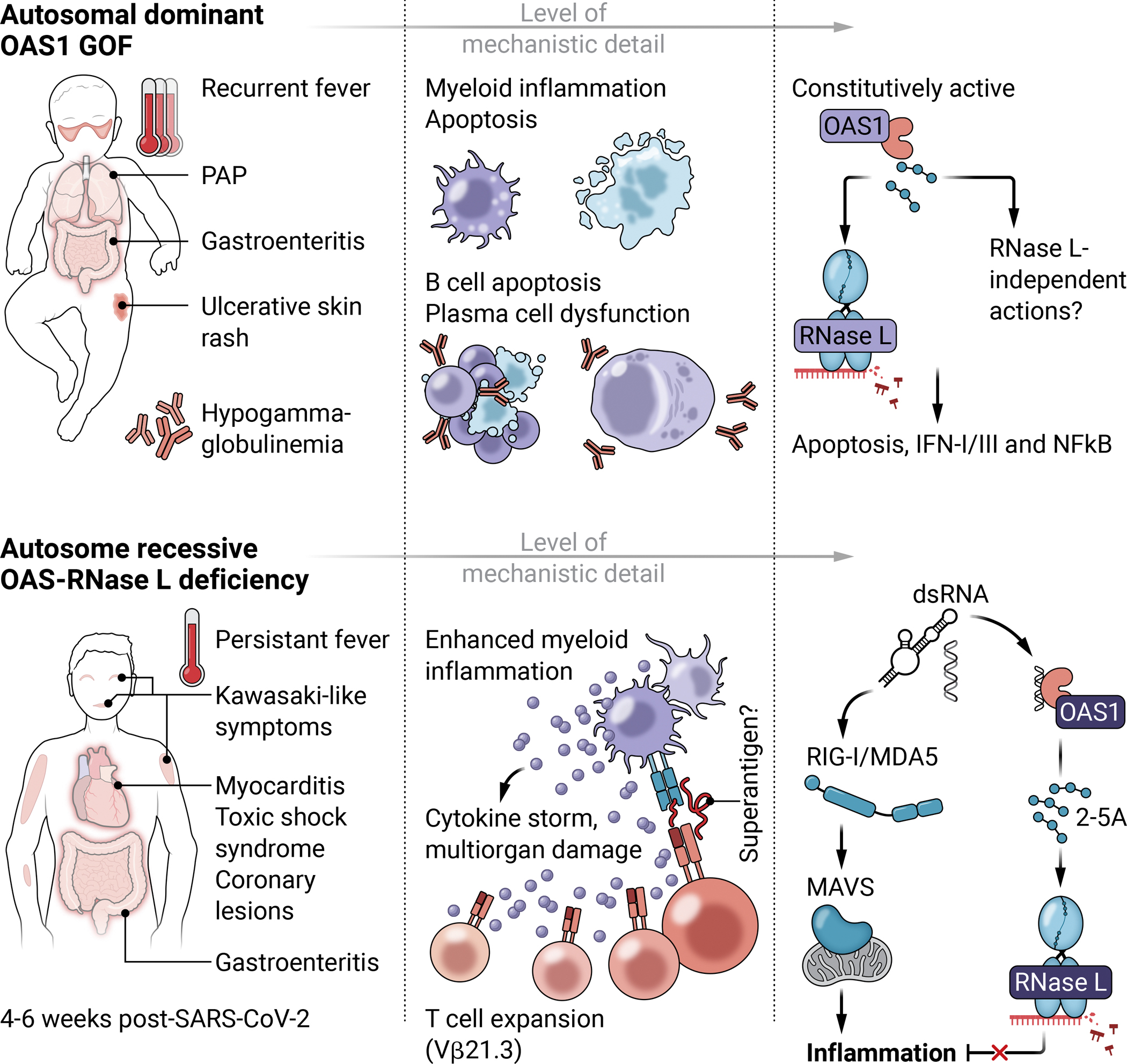
Human inborn errors of the OAS-RNase L pathway. The main clinical features of the currently known human diseases caused by inborn errors of the OAS-RNase L pathway are illustrated alongside the proposed cellular and molecular mechanisms. Germline gain-of-function mutations in OAS1 lead to constitutive production of 2–5A and thus constant activation of RNase L. The continuous generation of the degradation products from RNase L perpetuate the feed-forward loop of the inflammatory RIG-I/MDA5–MAVS pathway, but also proapoptotic pathways, especially in B cells and myeloid cells where basal level expression of OAS1 and RNase L are high. In autosomal recessive deficiencies of OAS1, OAS2, and RNase L, the restriction on MAVS-mediated inflammation in myeloid cells is lost, leading to excessive inflammation. PAP – pulmonary alveolar proteinosis.

Human OAS1 gain of function (GOF) was recently reported to underlie an autoinflammatory condition characterized by a combination of infantile-onset pulmonary alveolar proteinosis (PAP), hypogammaglobulinemia, and flares of autoinflammatory symptoms after infectious episodes, with recurrent fever, dermatitis, and inflammatory bowel disease accompanied by a flare-up of PAP (Fig. 2) (141). This condition was shown to be an autosomal dominant disorder with four disease-causing missense mutations (A76V, C109Y, V121G, and L198V) reported to date in 10 cases from eight pedigrees of four different ethnicities (14, 141). Except for three siblings with maternal somatic mosaicism for an OAS1 mutation, these cases were all sporadic, caused by *de novo* mutations. Studies with recombinant proteins showed that the disease-causing OAS1 mutants are biochemically GOF, producing excessive amounts of 2–5A independently of dsRNA stimulation in vitro (14). Spontaneous RNase L-dependent rRNA degradation and significant repression of protein translation were observed in resting primary monocytes and B cells but not T cells from patients and in a human cell line overexpressing OAS1-GOF mutants, demonstrating the occurrence of 2–5A-mediated RNA decay in these cells, even in the basal state (14). Basal levels of OAS1 expression are particularly high in monocytes/macrophages and B cells, and are further induced by IFNs, potentially accounting for the lineage-specific dysfunctions underlying the characteristic symptoms—PAP and hypogammaglobulinemia, respectively.

Lung surfactant homeostasis is normally maintained by alveolar macrophages in a granulocyte-macrophage colony-stimulating factor (GM-CSF)-dependent manner, while defects in this process result in typical PAP (142, 143). This disease may be inherited or acquired and results from surfactant deficiency, surfactant dysfunction, or an insufficient number or function of alveolar macrophages (142, 143). In macrophages differentiated from induced pluripotent stem cells (iPSCs) derived from patients with OAS1-GOF, stimulation with IFN-α resulted in enhanced apoptosis, weaker adhesion, scavenger receptor expression, and impaired phagocytosis. The peripheral monocytes of two OAS1-GOF patients (with the A72V or C109Y mutations) presented low levels of GM-CSF receptor expression, suggesting that an impaired GM-CSF response in alveolar macrophages may also contribute to the development of PAP in these individuals. IFN secretion due to viral infection may induce the expression of constitutively active OAS1, thereby increasing 2–5A production, in turn potentially amplifying RNase L-dependent IFN signaling (105, 144). Indeed, in some patients with OAS1 GOF, PAP appears to have been triggered by respiratory viral infections (141). Also, the numbers of monocytes and B-lineage cells gradually decreased during flare-ups, possibly reflecting the induction of OAS1 expression leading to RNase L-mediated apoptosis, whereas peripheral blood cell counts remained normal outside of these periods.

Intriguingly, hypogammaglobulinemia in OAS1-GOF patients occurred during the neonatal period, before any natural exposure to viruses. This presumably resulted from apoptosis and dysfunction due to IFN exposure during plasmacyte differentiation. Consistently, primary B cells from OAS1 GOF patients show impaired proliferation and differentiation into memory B cells and plasmablasts in vitro, with higher frequencies of pre-apoptotic cells. However, the exact molecular mechanism underlying hypogammaglobulinemia in these individuals has yet to be determined. Alternatively, RNase L-independent mechanisms of disease in patients with OAS1 GOF cannot be excluded. Interestingly, GOF of OAS2 in mice led to failure to lactate in otherwise healthy mice, coinciding with epithelial cell death, proliferation inhibition, and a robust IFN response in the mammary gland. GOF in the other human OAS proteins have yet to be found but could presumably lead to similar cellular anomalies as OAS1 GOF in different tissues and of different severities (145).

## OAS-RNase L loss-of-function genotypes in humans: SARS-CoV-2-triggered post-infectious inflammation

Inherited autosomal recessive deficiencies of OAS1, OAS2, or RNASEL were described in 2023 in five children from unrelated kindreds of four different ethnicities (15). They were identified through an unbiased analysis of whole-exome or whole-genome sequencing data for an international cohort of patients with SARS-CoV-2–related multisystem inflammatory syndrome in children (MIS-C). The mutations of *OAS1* and *RNASEL*, each found in one MIS-C patient, were stop-gain mutations that led to a complete deficiency of the protein. The *OAS2* mutations were demonstrated to be hypomorphic based on the reduced capacity of the mutant proteins to produce 2–5A and to induce RNase L–mediate RNA cleavage activity. All patients with OAS-RNase L deficiency were otherwise previously healthy and had normal blood cell counts outside of the MIS-C episode. Indeed, excluding the OAS1-deficient patient who was only one month old at the time, none of the patients had any significant history of infection despite having reached their preteens.

MIS-C is a severe acute hyperinflammatory syndrome that occurs almost exclusively in children (~1 case per 10,000 infected children, occurring at a median age of 8–9 years) four to six weeks after SARS-CoV-2 infection (146). Patients present with prolonged fever, mucosal erythema and skin rash, and gastrointestinal symptoms with biological signs of systemic inflammation with multiorgan lesions. MIS-C can be complicated by myocarditis, coronary artery lesions, or toxic shock syndrome requiring intensive care (Fig. 2). Immunological studies have consistently highlighted sustained myeloid activation and a cytokine storm in these patients (146). MIS-C patients have only mild or no symptoms during acute SARS-CoV-2 infection and typically do not present viral pneumonia. Consistently, in vitro infection of patient-derived dermal fibroblasts and A549 cells lacking OAS1, OAS2, or RNase L with SARS-CoV-2 showed that OAS1-, OAS2-, or RNase L-deficient cells restricted SARS-CoV-2 as efficiently as wild-type cells. However, in tests on peripheral blood mononuclear cells challenged with SARS-CoV-2, the myeloid cells of patients with deleterious biallelic variants of OAS-RNase L pathway components had higher levels of transcripts for proinflammatory cytokines than control cells. This hyperinflammatory phenotype was mirrored in monocytic cell lines and monocyte-derived dendritic cells with genetic deficiencies of OAS1, OAS2, or RNase L stimulated with poly(I:C), SARS-CoV-2, SARS-CoV-2–infected cells and their RNA. In THP-1 monocytic cells deficient for RNase L, hyperinflammation following poly(I:C) stimulation was attenuated by deletion of RIG-I or MDA5 and completely abolished by MAVS deletion.

**Figure 3.**
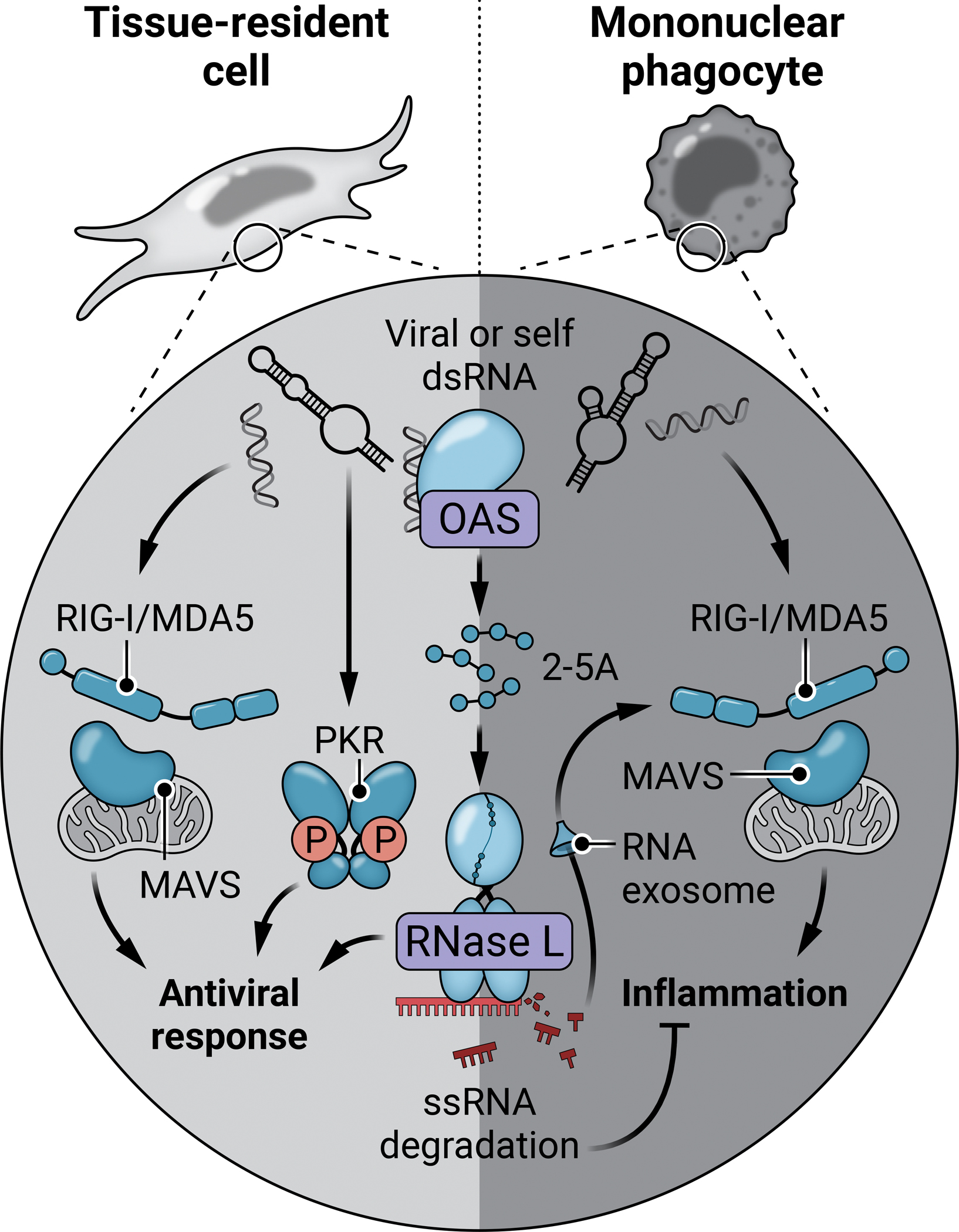
The OAS-RNase L pathway at the junction of infection and inflammation. The catalytically active OASs (OAS1, OAS2, OAS3) bind cytosolic double-stranded RNA of various origins (viral genome, viral replication intermediates, endogenous self-RNA or RNA from damaged cells), which then produce 2’-5’ oligoadenylates (2–5A) as secondary messengers, which activate the latent RNase L. Activated RNase L then cleaves single-stranded RNA of viral or host origin. The OAS-RNase L pathway contributes to the antiviral response in tissue-resident cells (e.g., epithelial cells, fibroblasts), whereas, in mononuclear phagocytes, it plays a key role in limiting the RIG-I/MDA5–MAVS-dependent inflammatory response.

Thus, in natural conditions, the human OAS-RNase L pathway appears to be redundant for the restriction of SARS-CoV-2 replication in the respiratory tract and probably for that of other common viruses, but crucial for regulation of the RIG-I/MDA5-MAVS–mediated inflammatory response to SARS-CoV-2 in mononuclear phagocytes (Fig. 3). Indeed, based on this observation, an enhanced inflammatory response leading to a cytokine storm may contribute to the association between the *OAS1* splice variant and severe COVID-19 found in the GWA studies (71, 126–129). Similarly, the inflammatory morbidity of certain viruses may be linked to their mechanisms for evading the OAS-RNase L pathway (e.g., MERS-CoV, influenza A, vaccinia virus) (89). Thus, unleashing MAVS-mediated inflammation may be a general innate immune safeguard against pathogens attempting to evade the OAS-RNase L pathway (147). Nevertheless, MIS-C remains unique in that it develops after the clinical resolution of acute infection.

Additional research is needed to understand which specific features of SARS-CoV-2 in relation to the OAS-RNase L pathway and the myeloid compartment give rise to a post-infectious hyperinflammatory syndrome. Further studies are also required to determine whether OAS-RNase L deficiency affects lymphocytes beyond their cytokine production (e.g., activation/quiescence, proliferation, apoptosis). Furthermore, we cannot exclude the possibility that OAS-RNase L deficiency leads to impaired SARS-CoV-2 restriction in other tissues, such as the gut epithelium or the myocardium, potentially resulting in atypical tissue damage or viral persistence. In any event, human inborn errors of immunity affecting the OAS-RNase L pathway have not only drawn attention to the importance of OAS-RNase L in controlling inflammation in mononuclear phagocytes in natura but have further highlighted the importance of homeostasis of the 2–5A system to the cell and the whole organism.

## Concluding remarks

The last 50 years have witnessed the expansion of research into the diverse roles of OASs, 2–5A and RNase L, most notably in host antiviral defense. Most human and mouse studies have focused on dissecting the mechanisms of action of these molecules as IFN-induced antiviral effectors. These studies have shed light on the biochemical function of each molecule and the broad impact of the pathway on host cellular and viral ssRNA degradation, translational arrest, and apoptotic cell death, focusing on the issue of how this pathway directly or indirectly exerts its antiviral effects. Investigations into the role of the OAS-RNase L pathway in humans under natural conditions remained largely unfruitful until recently. During the COVID-19 pandemic, GWAS consistently reported that common polymorphisms at the *OAS1/2/3* locus were associated with severe and critical COVID-19, the first demonstration of a role for this system in in natura human antiviral defenses. No inborn errors of the OASs or RNase L have been reported to underlie COVID-19 pneumonia. Surprisingly, later in the COVID-19 pandemic, recessive inborn errors of OAS1, OAS2 and RNase L were identified in children suffering from MIS-C, a postviral inflammatory syndrome occurring after benign respiratory tract infection with SARS-CoV-2. Contrary to the anticipated role of the OAS-RNase L pathway in defense against acute viral replication, epithelial cells and fibroblasts defective for this pathway restricted SARS-CoV-2 normally. Instead, the pathogenesis of MIS-C in affected children was explained by an exaggerated mononuclear phagocyte inflammatory response to SARS-CoV-2, SARS-CoV-2-infected cells, and their RNA. This, in turn, suggests that the main protective function of the OAS-RNase L pathway against SARS-CoV-2 may be the control of phagocyte-driven systemic inflammation during the later stages of infection, rather than the restriction of viral replication and spread through the respiratory tract at earlier stages.

Consistently, gain-of-function *OAS1* mutations in humans have been shown to result in autoinflammation driven by myeloid cells (14, 141). In line with these observations, autosomal dominant OAS2 deficiency was recently described in one patient who presented with autoimmune vasculitis marked by unusually severe autoinflammatory flares and coronary artery lesions, possibly triggered by a viral infection (37). The discovery of inborn errors of the OAS-RNase L pathway refocused attention on this pathway as a key player in the maintenance of inflammatory responses in mononuclear phagocytes, both in basal conditions and following viral infection. These findings have also confirmed the long-standing suspicions that the impact of the activation of the OAS-RNase L pathway is cell type-dependent (Fig. 3).

The possibility that recessive OAS-RNase L deficiency also affects antiviral responses in cells of other tissues injured during MIS-C, such as cardiomyocytes, enterocytes, and endothelial cells, cannot be excluded. Deficiencies of this pathway may have resulted in sustained SARS-CoV-2 replication in these cells before the triggering of monocytic hyperinflammation. Whether other viruses not yet encountered by the identified OAS-RNase L-deficient patients will elicit different phenotypes, and how the immune responses of OAS-RNase L-deficient individuals will change with age, remains to be seen. As of now, many details of the OAS-RNase L pathway remain elusive and warrant further investigation. Nevertheless, OAS1, OAS2 and RNase L are each essential for the regulation of immunity to SARS-CoV-2 in a given cell type, as genetic deficiency of any of these molecules results in the same immunological and clinical phenotype in humans. Over the course of evolution, selection has resulted principally in diversity of the OASs rather than RNase L. This raises questions about the possible non-redundancy of the catalytically active OASs despite their common origin and their structural and functional similarities. The roles of OAS3 and OASL in humans in natura remain unknown. Both the genes encoding these molecules are strongly expressed in myeloid cells, like the genes encoding OAS1, OAS2 and RNase L. We can, therefore, speculate that inborn errors of OAS3 and OASL may underlie MIS-C in other patients, and/or other forms of Kawasaki disease triggered by other viruses (146). Human OASL, which functions in an RNase L-independent manner, may also be a crucial antiviral factor under natural conditions of infection, like the RNase L-independent Oas1b and Oasl1/2 in mice in vivo. Future studies aiming to identify human inborn errors of the OASs and RNase L in infections and in postinfectious inflammatory conditions will further our understanding of the role of these important host defense genes in natural conditions of infection.

More generally, the finding that recessive deficiencies of a classic ‘antiviral effector’ pathway underlie a postviral inflammatory syndrome provides a key point for reflection concerning the nature of ‘viral inflammation’. At least two waves of acute inflammation with different mechanisms may occur following infection with a given virus in genetically vulnerable individuals: inflammation driven by acute viral replication (acute viral inflammation, such as COVID-19 pneumonia) and postviral inflammation (such as MIS-C) (Fig. 2). In the case of SARS-CoV-2 infection, inborn errors of type I IFN immunity and autoantibodies against type I IFNs underlie severe COVID-19 pneumonia due to unrestricted viral production in the lungs, whereas inborn errors of the OAS-RNase L pathway underlie post-SARS-CoV-2 MIS-C due to unchecked inflammation mediated by RIG-I/MDA5–MAVS in mononuclear phagocytes. Could different mechanisms of inflammation underlie acute viral inflammation and postviral inflammation? And what of the many other pathogenic human viruses that have never been subject to intensive investigations like those performed for SARS-CoV-2? Further investigations will be required to clarify this point.

Importantly, defective type I IFN immunity due to inborn errors of immunity or autoantibodies has increasingly being found to underlie severe acute inflammation in specific organs in response to various viruses (e.g. SARS-CoV-2 and influenza pneumonia, herpes simplex encephalitis, West Nile virus encephalitis). Conversely, excessive type I IFN immunity due to a GOF of key ISGs (e.g. *STING*, *OAS1, IFIH1*, *RIGI*) has also been shown to be related to inflammatory conditions (i.e. interferonopathies). The OAS-RNase L pathway appears to occupy a key position in these cascades of immunity to infection and inflammation, helping to maintain a delicate balance between these two states. Recently acquired knowledge in this field may also help to resolve other enigmatic autoimmune or autoinflammatory postinfectious conditions in humans such as classic Kawasaki disease, acute disseminated encephalomyelitis, rheumatic fever, and Henoch-Schönlein purpura. Identification of both the host genetic causes and the infectious triggers should transform our understanding of the pathogenesis of such conditions and guide future strategies for their treatment.

## Supplementary Material

Table S1 Table S1. The antiviral effects of the OAS-RNase L pathway in vitro.
