# Supplementary material for: The OAS-RNase L pathway: insights from experiments of nature
Source: Sci Immunol. Author manuscript; Available in PMC 2026 Feb 20. (PMC12922763; doi:10.1126/sciimmunol.ads9407)
Supplement: Table S1 — The antiviral effects of the OAS-RNase L pathway in vitro. [file NIHMS2138343-supplement-Table_S1.docx]

**Table S1. The antiviral effects of the OAS-RNase L pathway in vitro.**

Summary of the antiviral capacity of human and mouse OASs and RNase L tested in vitro. The effect of RNase L deficiency in mice in vivo or in vitro with cells derived from RNase L KO mice have been reviewed extensively in Silverman 2007 (67). Viral mechanisms of evading the OAS-RNase L pathway have been reviewed extensively in Drappier et al., 2015 (89) and Silverman 2007 (67). Table is adapted and expanded from Silverman 2007 (67).
